# Executive Function and Language Expression in Preschool Children: A Random Intercept Cross-Lagged Panel Study

**DOI:** 10.3390/jintelligence14080165

**Published:** 2026-08-01

**Authors:** Jin-Long Liang, Chin-Hsuan Hsieh, Lung-An Shen, Hsin-Chen Lu, Ho-Tang Wu, Xin-Ling Cui

**Affiliations:** 1 College of Education Science, Northwest Normal University, Lanzhou 730070, China; liangjl08@163.com (J.-L.L.); cuixl08@163.com (X.-L.C.); 2 Department of Education, National Kaohsiung Normal University, Kaohsiung 82444, Taiwan; chinhsuan0914@gmail.com (C.-H.H.); 811351101@mail.nknu.edu.tw (H.-C.L.); 3 Department of Early Childhood Care and Education, Cheng-Shiu University, Kaohsiung 83347, Taiwan; 8116@gcloud.csu.edu.tw

**Keywords:** executive function, language expression, preschool children, random intercept cross-lagged panel model

## Abstract

This study examined longitudinal between-child and within-child associations between executive function (EF) and language expression (LE) in preschool children. Data were drawn from four waves of the Kids in Taiwan: National Longitudinal Study of Child Development and Care at 36, 48, 60, and 72 months (*N* = 1694). Longitudinal measurement invariance was examined before estimating a random intercept cross-lagged panel model (RI-CLPM), which separated stable between-child differences from within-child temporal fluctuations. EF and LE showed temporal continuity and positive concurrent associations. At the within-child level, occasions on which children displayed stronger LE than their own typical level consistently preceded relatively stronger EF one year later, whereas the reverse pathway was not consistently supported. These findings refine language-based accounts of self-regulation by suggesting that children’s everyday use of language to answer, explain, clarify, and organize information may be relevant to subsequent regulatory functioning. Practically, language-supported activities involving rule explanation, self-guidance, and reflective dialogue may be useful components of early EF support, although causal conclusions are not warranted from this observational study.

## 1. Introduction

The preschool years are a period of substantial development in executive function (EF) and language expression (LE), both of which contribute to children’s learning, social participation, and self-regulation (Best & Miller, 2010; Broomell & Bell, 2022; Diamond, 2013). EF broadly refers to top-down processes involved in maintaining goals, inhibiting dominant responses, and flexibly adjusting behavior (Carlson et al., 2013; Diamond, 2013). Because the present study relies on caregiver reports rather than performance-based tasks, EF was operationalized more narrowly as a brief index of everyday regulatory behavior. The items reflect behavioral inhibition, sustained regulation following reminders, organization and rule following, awareness of behavioral consequences, and engagement in cognitively demanding activities. Thus, this measure should not be interpreted as a direct assessment of distinct EF components such as working memory, inhibitory control, and cognitive flexibility.

Within the same everyday contexts, language may provide children with an additional means of organizing experience and regulating action. In this study, LE refers specifically to caregiver-observed expressive-language behaviors. Expressive language involves children’s production and functional use of words, sentences, and discourse to communicate meanings and intentions (Bates et al., 1991; Shokrkon & Nicoladis, 2022). The nine items assess descriptive word use, responses to what and why questions, production of connected or complex utterances, reference to absent people or events, conversational initiation and repair, and explanation of procedures or rules. Accordingly, the index samples selected expressive, syntactic, pragmatic, and discourse-related behaviors rather than overall language competence. Because language measures differ in whether they assess vocabulary, receptive or expressive language, syntax, pragmatics, or broader competence, findings must be interpreted in relation to the specific domain and measurement approach used (Bruce & Bell, 2022; Shokrkon & Nicoladis, 2022). The present index therefore should not be regarded as a comprehensive assessment of receptive language, vocabulary breadth, phonology, or narrative quality.

### 1.1. Developmental and Directional Relations Between EF and LE

Having clarified the scope of the two constructs, the next question concerns how they unfold and relate across early childhood. Both EF and LE show developmental continuity during this period. Earlier EF predicts later EF performance, although preschool EF remains highly plastic and responsive to maturation and experience (Best & Miller, 2010; Broomell & Bell, 2022). Expressive-language skills likewise develop cumulatively as children acquire words and sentence structures and use language across increasingly varied communicative situations (Bates et al., 1991; Huttenlocher et al., 2010; Rowe, 2012). These parallel developmental patterns provide a basis for examining autoregressive continuity in both constructs from 36 to 72 months.

One possible direction of influence is from EF to LE. EF may support expressive-language behavior by helping children maintain relevant information, inhibit competing responses, select words, integrate sentence elements, and adjust an utterance to contextual demands (Baddeley, 2003; Filipe et al., 2023; White et al., 2017). Consistent with this account, verbal working memory and cognitive flexibility contribute to receptive and expressive language, while cognitive control is associated with the production of contextually appropriate referential expressions (Filipe et al., 2023; Serratrice & De Cat, 2020). Therefore, the expectation that children use EF-related processes to produce clearer and more contextually appropriate language is grounded in prior theory and evidence, although these mechanisms were not directly tested in the present study.

The relationship may also operate in the opposite direction. LE may support EF by giving children linguistic tools for representing rules, labeling goals, planning steps, and monitoring actions. Sociocultural and private-speech accounts propose that externally directed language is gradually internalized and used for self-guidance and behavioral regulation (Vygotsky, 1978; Winsler et al., 2009). In line with this perspective, partly internalized private speech has been linked to cognitive flexibility, and longitudinal research has shown that early expressive-language growth predicts subsequent EF trajectories (Alarcón-Rubio et al., 2014; Kuhn et al., 2016). The present LE measure did not directly assess private or inner speech; these processes are therefore treated as theoretically plausible explanations rather than mechanisms measured in this study.

Despite support for both pathways, prior findings do not establish a single direction of influence. Some studies emphasize language-to-EF relations, including evidence that expressive-language growth predicts later EF (Kuhn et al., 2016) and that language predicts subsequent EF and theory of mind (Shahaeian et al., 2023). Other studies show that EF components explain variation in language outcomes (Filipe et al., 2023) or report EF-to-language associations in cross-lagged analyses (Zhu et al., 2025), whereas still others support reciprocal relations (Slot & von Suchodoletz, 2018). Reviews likewise identify language-to-EF, EF-to-language, and bidirectional patterns, suggesting that differences in age, language domain, measurement approach, and statistical model may contribute to the inconsistent conclusions (Bruce & Bell, 2022; Shokrkon & Nicoladis, 2022).

These mixed findings make it important to distinguish stable differences between children from developmental fluctuations within the same child. The central theoretical question is not only whether children who generally display stronger LE also tend to display stronger EF, because such between-child associations may reflect enduring differences in maturation, family language input, parental education, or learning opportunities. A more stringent question is whether a child’s temporary elevation above his or her own typical LE level precedes subsequent changes in EF, or vice versa. The random intercept cross-lagged panel model (RI-CLPM) is well suited to this question because it separates within-child temporal dynamics from stable between-child differences (Hamaker et al., 2015). This distinction allows language-supported self-regulation and EF-supported language production to be evaluated at the level at which change occurs within individual children.

### 1.2. Purpose of the Present Study and Research Questions

Building on this distinction, the present study used four-wave longitudinal data from Taiwan to examine the stability, concurrent association, and within-child directional relations between caregiver-reported EF and LE from 36 to 72 months. Rather than treating LE as a comprehensive measure of language competence, the study focuses on a brief survey-based index of productive and communicative behaviors observed in daily life. Accordingly, the following three research questions were addressed:

1. Do caregiver-reported EF and LE show cross-time autoregressive effects?
2. Are EF and LE concurrently associated at the same time point?
3. Do the two constructs show cross-time within-child cross-lagged effects?

## 2. Materials and Methods

### 2.1. Participants

The sample for this study was drawn from the Kids in Taiwan: National Longitudinal Study of Child Development and Care (KIT). Children with tracking records at 36, 48, 60, and 72 months were first selected to ensure that all four waves of longitudinal data corresponded to the same individuals. After data merging, the final analytic sample included 1694 children. Further inspection of item-level missing values showed that each wave of the EF and LE items had 2 to 7 missing responses, totaling 123 missing item responses across the four waves, corresponding to a missing rate of 0.12%. Therefore, full information maximum likelihood (FIML) in Mplus 9.1 was used to handle partial missing data in subsequent model estimation. FIML uses all available observed information for parameter estimation without deleting cases with partial missing data, thereby reducing sample loss and potential estimation bias associated with traditional deletion methods (Little & Rubin, 2019).

The demographic characteristics of the final analytic sample were summarized based on SPSS frequency distribution results and included child gender, respondent’s relationship to the child, and parental education level (see Table 1).

### 2.2. Measures

The main variables were drawn from the KIT secondary dataset. Both scales were developed for Taiwanese children aged 36 to 72 months, and the draft questionnaires underwent expert review, cognitive interviews, pilot testing, and pretesting. The measures were selected because they provide repeated, age-consistent observations of behaviors that occur across natural daily situations, which is advantageous for a four-wave population-based study.

The six-item EF Scale was treated as a brief caregiver-reported index of EF in everyday contexts. Its items concern awareness of behavioral consequences, slowing and improving behavior after reminders, engagement in cognitive or puzzle activities, returning objects to their usual place, lowering and maintaining voice volume when requested, and stopping and maintaining inhibition of running or jumping when told “No.” These items reflect everyday behavioral regulation and organization but do not yield separate performance-based scores for working memory, inhibitory control, or cognitive flexibility. 

The nine-item LE scale was treated as an index of expressive-language behaviors. Its content includes descriptive word production, answering what and why questions, producing complex or connected sentences, referring to absent people or events, initiating topics, requesting clarification, and explaining procedures or rules. These content groupings describe the items and are not separately validated subscales. The LE measure does not directly assess receptive language, standardized vocabulary breadth, phonological processing, narrative macrostructure, or overall language competence.

According to the KIT database construction documents, pretest internal consistency was high (Cronbach’s α = 0.95 for EF and 0.93 for LE). Both scales used a 4-point response format (1 = not at all able, 2 = barely able, 3 = fairly skilled, and 4 = very skilled) and were completed by the primary caregiver at each wave. Caregiver reports were appropriate for capturing repeated behavior across routines, interactions, and rule-based situations that may be difficult to observe in a single standardized session. Nevertheless, they represent caregiver perceptions and may be affected by rater expectations and shared-method variance; these limitations are considered in the Discussion. The same items were administered at all four waves, and total scores were used as observed indicators. Higher scores indicated more frequent or skilled EF and LE. Complete item wording is provided in Appendix A.

### 2.3. Analytic Strategy

#### 2.3.1. Measurement Invariance

Longitudinal measurement invariance was examined to evaluate whether the item-factor relations remained sufficiently comparable over time (Cheung & Rensvold, 2002; Putnick & Bornstein, 2016). Confirmatory factor analysis included EF and LE in the same model. Configural, metric, scalar, and residual invariance were tested sequentially. Metric invariance was particularly relevant to interpreting longitudinal associations, whereas scalar and residual invariance were required for stronger claims about latent mean or residual equivalence.

Model fit was evaluated using the chi-square statistic (χ^2^), comparative fit index (CFI), incremental fit index (IFI), root mean square error of approximation (RMSEA), and standardized root mean square residual (SRMR). According to common criteria, CFI and IFI values of 0.90 or above indicate acceptable model fit, whereas values of 0.95 or above indicate good fit; RMSEA and SRMR values below 0.10 and 0.08, respectively, indicate acceptable fit (Hu & Bentler, 1999; Kline, 2023).

Nested models were then examined to test configural, metric, scalar, and residual invariance using multigroup analysis in AMOS 28.0 (IBM Corp., 2021, Armonk, NY, USA). Finally, ΔCFI was used to determine whether measurement invariance was supported. Cheung and Rensvold (2002) noted that CFI is commonly used and appropriate for evaluating measurement invariance in large samples. Chen (2007) proposed a ΔCFI criterion of less than 0.01, which was adopted in the present study.

#### 2.3.2. Descriptive Statistics and Correlations

In structural equation modeling, the means and standard deviations of variables are reported to describe overall sample performance and variability, thereby supporting the robustness of subsequent model estimation. Pearson product–moment correlations were also computed to examine the associations among variables. SPSS 31.0 was used to calculate the means, standard deviations, and Pearson correlations of EF and LE scale scores at each wave (IBM Corp., 2025).

#### 2.3.3. Cross-Lagged Analysis

This study used an RI-CLPM to examine the longitudinal relationship between EF and LE at 36, 48, 60, and 72 months. RI-CLPM distinguishes stable between-person differences from within-person temporal fluctuations, thereby avoiding the traditional cross-lagged panel model’s tendency to conflate stable between-child differences with within-child longitudinal effects. In the model, random intercept factors were specified for EF and LE (RI_EF and RI_LE). Each random intercept factor was indicated by four observed indicators from the four time points, with factor loadings fixed to 1 to represent stable individual differences in EF and LE.

**Figure 1:**
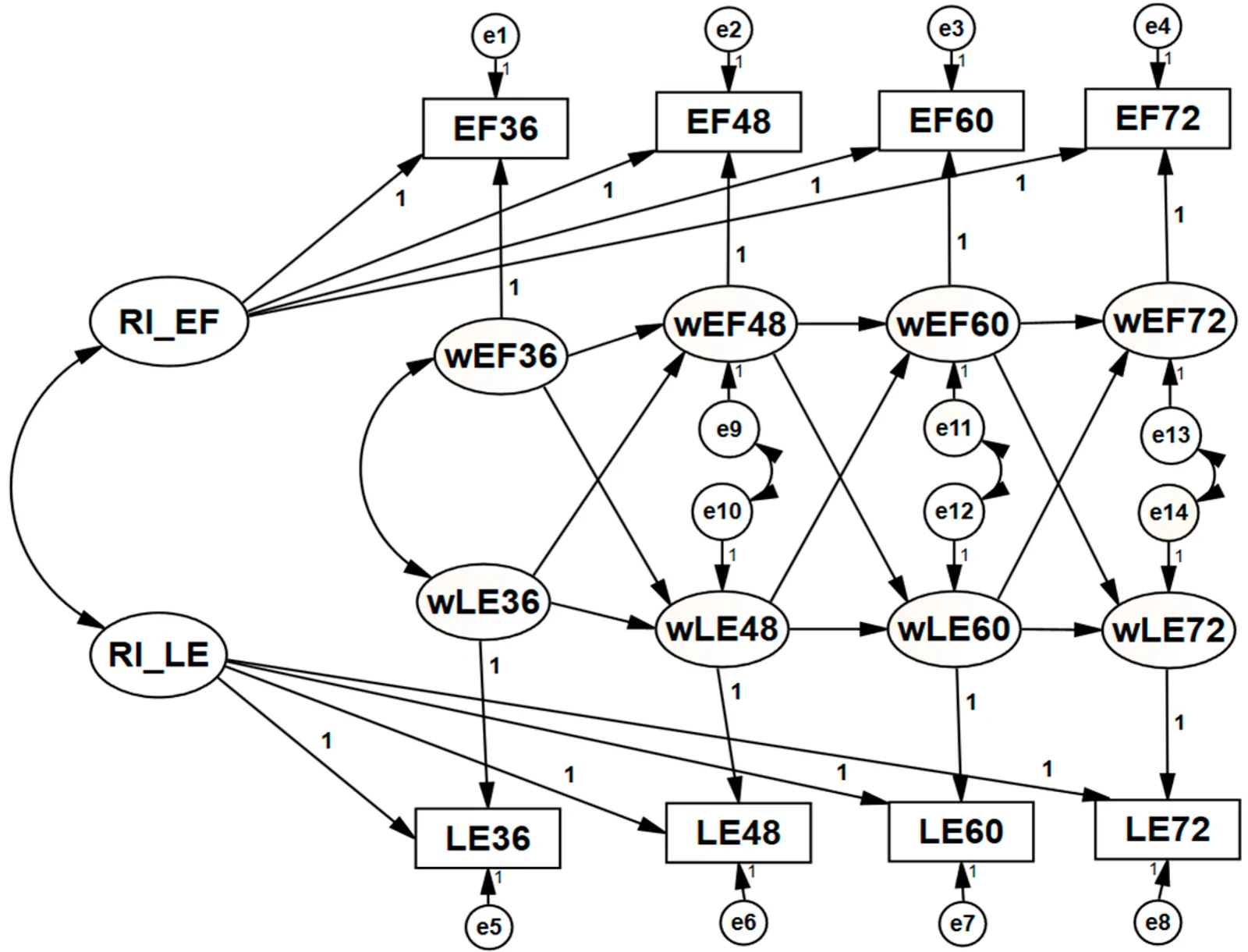
Hypothesized RI-CLPM. Note: EF = executive function; LE = language expression. RI_EF = random intercept of EF; RI_LE = random intercept of LE; numbers indicate age in months; wEF = within-person EF; wLE = within-person LE; e1 to e14 are residual terms. Single-headed arrows represent directional regression paths, whereas double-headed arrows represent covariances or correlations between variables.

At the within-person level, latent within-person factors were specified for each time point, including wEF36, wEF48, wEF60, wEF72, and wLE36, wLE48, wLE60, and wLE72. These factors represented each child’s deviation from his or her own average level at a given age. The model estimated three types of paths: (a) autoregressive paths, which tested whether EF or LE at one time point predicted the same construct at the next time point; (b) concurrent associations, which tested the association between EF and LE at the same time point; and (c) within-child cross-lagged paths, which tested whether EF at one time point predicted LE at the next time point and whether LE at one time point predicted EF at the next time point. The model also estimated the correlation between RI_EF and RI_LE to examine the association between the two constructs at the stable between-child level (see Figure 1).

RI-CLPM was estimated in Mplus Version 9.1 (Muthén & Muthén, 2025) using maximum likelihood (ML) estimation. For partial missing data, FIML was used to estimate model parameters based on all available observed information. Results are reported as standardized coefficients and their 95% confidence intervals. Confidence intervals were taken directly from the lower 2.5% and upper 2.5% estimates in the Mplus output.

## 3. Results

### 3.1. Measurement Invariance

The invariance tests presented in Table 2 supported configural and metric invariance for both EF and LE (ΔCFI = 0.004), indicating broadly comparable factor structures and item–factor relations across the four waves. For the EF model, the modification index for the covariance between residuals e11 and e12 was 151.43; therefore, this residual covariance was freely estimated and retained across the subsequent invariance models. Allowing these residuals to covary indicates that the two observed indicators shared additional item-specific variance beyond that explained by the latent EF factor, possibly because of similarity in item content, wording, or measurement context. Scalar and residual invariance were not supported. Consequently, observed or latent mean differences were not interpreted as evidence of true developmental growth, and the longitudinal path findings were interpreted cautiously within the limits of metric invariance.

### 3.2. Means, Standard Deviations, and Correlations of EF and LE

Table 3 shows moderate cross-time continuity within each construct and positive concurrent correlations between EF and LE at all four ages. Cross-time correlations between early EF and later LE, and between early LE and later EF, were also generally positive.

Observed mean scores increased with age. Because scalar invariance was not established, these descriptive trends were not interpreted as latent developmental growth; subsequent conclusions focused on autoregressive, concurrent, and within-child cross-lagged associations.

### 3.3. Random Intercept Cross-Lagged Effects Between EF and LE

#### 3.3.1. Overall Model Fit

The RI-CLPM showed acceptable-to-good fit (χ^2^(11) = 125.18, *p* < .001, RMSEA = 0.08, 90% CI [0.07, 0.09], CFI = 0.98, TLI = 0.96, and SRMR = 0.08). The significant chi-square was interpreted in light of its sensitivity to the large sample, while the remaining indices indicated that the model reproduced the observed covariance structure adequately.

#### 3.3.2. Random Intercept Cross-Lagged Effects

Table 4 reports the complete standardized estimates and confidence intervals. At the within-child level, autoregressive carryover was modest for EF (β = 0.09–0.25) and stronger for LE (β = 0.42–0.45). EF and LE covaried positively within each wave (β = 0.42–0.50).

**Figure 2:**
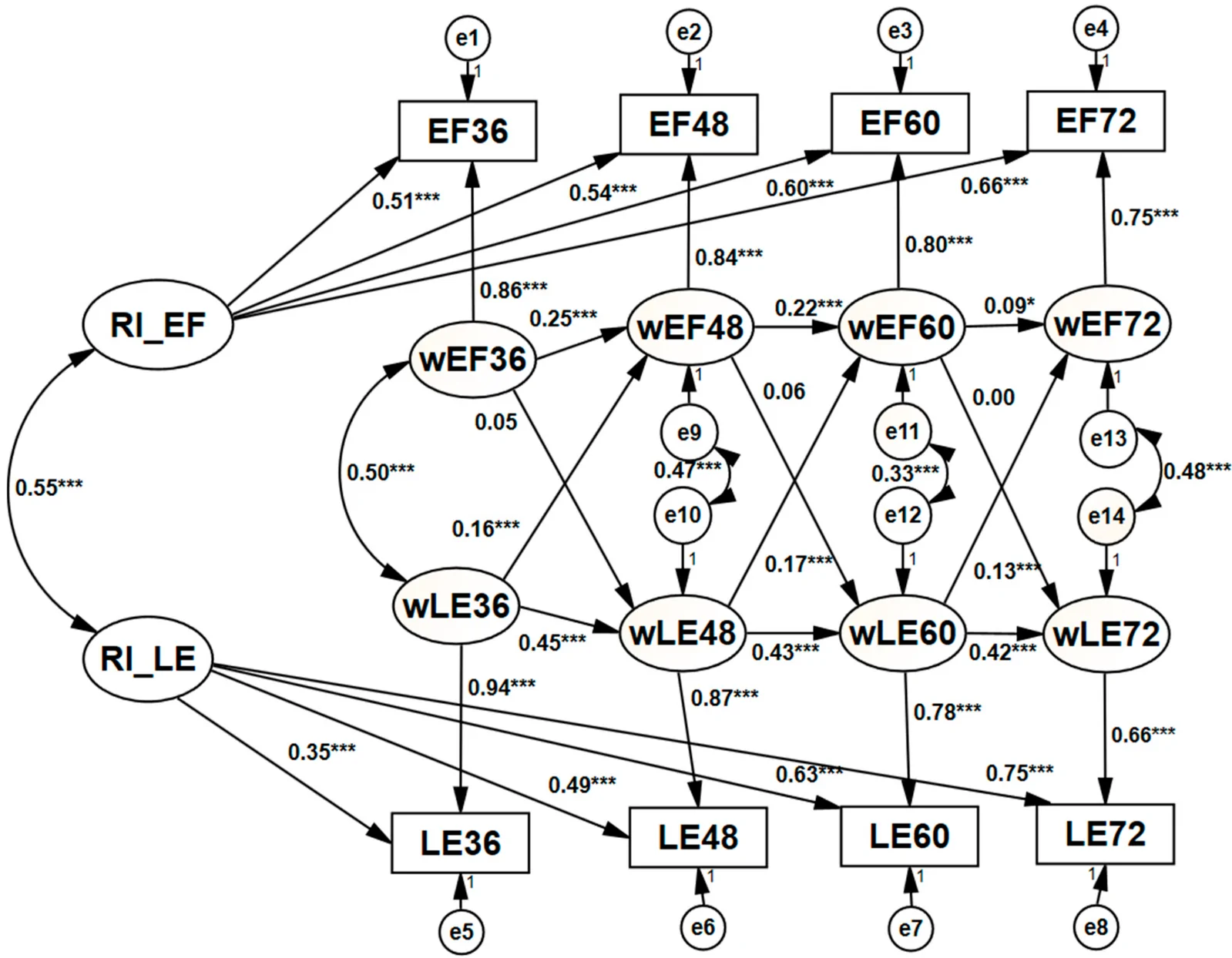
Random intercept cross-lagged effects between EF and LE. Note: EF = executive function; LE = language expression. Numbers indicate age in months. RI_EF = random intercept of executive function; RI_LE = random intercept of language expression. Single-headed arrows represent directional regression paths, whereas double-headed arrows represent covariances or correlations between variables.* *p* < .05. *** *p* < .001.

The principal cross-lagged pattern was asymmetric. Occasions on which children showed higher LE than their own typical level predicted relatively higher EF at the next wave (β = 0.16–0.33, all *p* < .001). In contrast, EF-to-LE paths were small (β = 0.00–0.06) and nonsignificant. At the stable between-child level, the random intercepts were positively correlated (β = 0.55, *p* < .001) (see Figure 2), indicating that children with higher EF also tended to show higher LE.

## 4. Discussion

The main contribution of this study is not simply the finding that children with stronger language tended to have stronger EF. By separating stable between-child differences from within-child fluctuations, the RI-CLPM showed that the prospective association was asymmetric within children: higher-than-usual LE preceded relatively stronger EF, whereas the reverse pathway was not consistently observed. This pattern helps refine theories of language-supported self-regulation while also showing why associations derived from cross-sectional or traditional cross-lagged models should not automatically be interpreted as within-child developmental processes.

### 4.1. Measurement Comparability and Developmental Continuity

Metric invariance indicated that the EF and LE items related to their respective constructs in broadly comparable ways across 36 to 72 months. Because scalar and residual invariance were not supported, the study appropriately avoided strong interpretations of mean-level growth. Within these limits, both constructs showed temporal carryover. LE displayed stronger autoregressive continuity than EF, which may reflect the cumulative nature of productive language experience, whereas the weaker later EF carryover is consistent with continuing plasticity in preschool regulatory behavior (Best & Miller, 2010; Broomell & Bell, 2022). These patterns concern the caregiver-reported indices used here and should not be generalized to all components of EF or language competence.

### 4.2. Language Expression as a Potential Support for Self-Regulation

The LE-to-EF pathway is consistent with sociocultural and private-speech accounts in which language becomes a tool for representing rules, maintaining goals, sequencing actions, and monitoring responses. A child who can answer why questions, explain procedures, request clarification, or connect clauses may have more readily available linguistic resources for making task demands explicit (Alarcón-Rubio et al., 2014; Vygotsky, 1978; Winsler et al., 2009). Such behavior could support self-reminding and action monitoring when the child must wait, switch rules, or complete a multistep task.

This interpretation is compatible with longitudinal evidence that expressive-language growth predicts later EF trajectories (Kuhn et al., 2016) and with findings linking partly internalized private speech to cognitive flexibility (Alarcón-Rubio et al., 2014). However, the present LE scale assessed outward expressive behaviors rather than private or inner speech. The proposed self-regulatory processes therefore remain plausible mechanisms, not mechanisms directly demonstrated by the current data. Experimental or intensive observational studies are needed to determine whether increases in these expressive behaviors lead to greater use of verbal self-guidance.

### 4.3. Why EF Did Not Consistently Predict Later LE

The absence of consistent EF-to-LE paths does not imply that EF is irrelevant to language. Prior studies have found EF associations with receptive and expressive language or with specific discourse demands (Filipe et al., 2023; Serratrice & De Cat, 2020; Zhu et al., 2025). One explanation is that some previously observed effects reflect stable between-child differences in maturation, home language input, parental education, or learning opportunities. Once RI-CLPM removed these enduring differences, little evidence remained that a temporary elevation in EF produced a one-year-later increase in the broad daily-language index.

A second possibility concerns measurement specificity. EF may be especially relevant to demanding language tasks such as complex narrative planning, referential choice, or resolving competing linguistic representations, whereas the nine-item total score combined several everyday behaviors with different cognitive demands. Aggregation may therefore attenuate component-specific EF effects. Third, both variables were reported by the same caregiver, which may strengthen concurrent covariance through shared perceptions without producing parallel cross-time effects. Finally, a 12-month interval may miss shorter-term EF influences that operate during moment-to-moment language production. These explanations are provisional and should be tested using task-based EF measures, standardized language assessments, multiple informants, and shorter assessment intervals.

### 4.4. Theoretical and Practical Implications

Theoretically, the findings distinguish two propositions that are often conflated: stable child differences in language and EF were moderately associated, but within-child temporal change was more consistently aligned with language-to-EF than EF-to-language prediction. This added value arises from testing change at the individual level rather than inferring developmental direction from differences between children. The results therefore lend qualified support to accounts in which productive language contributes to the organization of self-regulated action.

Practically, language-rich self-regulation activities may be worth incorporating into preschool routines. During turn-taking, an adult can encourage a child to verbalize, “It is not my turn yet; I will wait.” In a rule-switching game, children can restate the new rule, such as “Before we sorted by color; now we sort by shape.” During a multistep task, they can describe the sequence before acting. Shared reading, story retelling, role-play, and reflective dialogue can similarly invite children to explain goals, rules, strategies, and corrections. These examples illustrate possible applications rather than proven intervention effects; experimental studies are needed before concluding that such practices cause improvements in EF.

### 4.5. Limitations and Future Directions

Several limitations qualify the findings. First, RI-CLPM improves separation of stable between-child differences from within-child temporal associations, but the observational design cannot rule out time-varying confounding or establish causality. The cross-lagged paths should therefore be interpreted as prospective associations rather than causal effects.

Second, both constructs were assessed through brief caregiver reports. The EF index reflected everyday regulation-related behaviors rather than separate laboratory measures of working memory, inhibition, and cognitive flexibility (Carlson et al., 2013; Diamond, 2013). The LE index sampled selected productive, syntactic, pragmatic, and discourse-related behaviors but did not comprehensively assess receptive language, vocabulary breadth, phonology, narrative macrostructure, or overall language competence (Bruce & Bell, 2022; Shokrkon & Nicoladis, 2022). Findings should therefore be generalized only to the forms of everyday behavior represented by these items.

Third, the use of the same caregiver for both scales creates possible shared-method variance and rater bias, especially for concurrent associations. Multimethod studies combining caregiver and teacher reports, direct EF tasks, standardized language assessments, and naturalistic observations would provide stronger construct validation and help determine whether the asymmetric cross-lagged pattern replicates across methods.

Fourth, only configural and metric invariance were supported. Although this permits cautious interpretation of longitudinal relations, scalar and residual noninvariance precludes strong conclusions about true mean growth and may indicate developmental changes in item thresholds or residual variation. Future studies should examine partial or approximate invariance and test whether specific items function differently across age.

Fifth, unmeasured contextual factors—including home language input, parent–child interaction, parental education, preschool experiences, teacher scaffolding, and peer interactions—may influence both constructs or condition their association (Balade et al., 2025; Huttenlocher et al., 2010; Rowe, 2012; Vygotsky, 1978). Future RI-CLPMs should incorporate such time-varying covariates, mediators, or moderators. More frequent measurement would also help evaluate whether EF influences language over shorter intervals than the one-year lags used here.

Finally, the sample was drawn from a Taiwanese Mandarin-speaking context. Language structure, family socialization, and preschool practices may shape how verbal expression is used for behavioral regulation. Cross-linguistic and cross-cultural replication is needed to establish whether the observed within-child LE-to-EF pattern is general or context-dependent.

## 5. Conclusions

This four-wave study shows that the association between executive function and language in early childhood is not explained solely by stable differences between children. At the within-child level, higher-than-usual caregiver-reported LE preceded relatively stronger EF, whereas the reverse pathway was not consistently observed. The findings refine language-based accounts of self-regulation by suggesting that children’s everyday use of language to answer, explain, clarify, and organize information may be relevant to later regulatory functioning. Because both constructs were measured with brief caregiver-report indices, the results concern everyday expressive and regulatory behaviors rather than comprehensive language competence or performance-based EF, and they do not establish causality. Future experimental, multimethod, and cross-cultural research is needed to test the proposed mechanisms and educational applications.

## Figures and Tables

**Table 1:** Demographic characteristics of the participants (*N* = 1694).

Variable	Category	*n*	%
Child gender	Boy	864	51.0
	Girl	830	49.0
Respondent’s relationship to child	Biological mother	1391	82.1
	Biological father	264	15.6
	Others	39	2.3
Father’s education level	Junior high school or below	98	5.8
	Senior high/vocational school	464	27.4
	Junior college	252	14.9
	University/two-year technical college	573	33.8
	Master’s degree or above	278	16.4
	Missing/other	29	1.7
Mother’s education level	Junior high school or below	97	5.7
	Senior high/vocational school	417	24.6
	Junior college	271	16.0
	University/two-year technical college	695	41.0
	Master’s degree or above	186	11.0
	Missing/other	28	1.7

Note: Percentages are based on the final analytic sample of 1694 children. Missing/other categories include participants with missing information or responses not classified into the listed educational categories.

**Table 2:** Measurement invariance results for EF and LE.

Model	χ^2^	*df*	RMSEA	SRMR	IFI	CFI	ΔCFI
EF							
Configural	1717.05 ***	227	0.06	0.04	0.91	0.907	
Metric	1795.51 ***	242	0.06	0.05	0.90	0.903	0.004
Scalar	4003.72 ***	260	0.09	0.10	0.77	0.766	0.137
Residual	5724.38 ***	278	0.18	0.11	0.66	0.659	0.105
LE							
Configural	3612.25 ***	561	0.06	0.05	0.92	0.916	
Metric	3784.96 ***	585	0.06	0.05	0.91	0.912	0.004
Scalar	5503.03 ***	610	0.07	0.07	0.87	0.865	0.047
Residual	16,735.66 ***	637	0.12	0.14	0.56	0.556	0.309

Note: ΔCFI is the absolute difference between two consecutive models. *** *p* < .001.

**Table 3:** Means, standard deviations, and correlation matrix for EF and LE (*N* = 1694).

	1	2	3	4	5	6	7	8
1. EF36	-							
2. EF48	0.53 ***	-						
3. EF60	0.45 ***	0.55 ***	-					
4. EF72	0.36 ***	0.49 ***	0.56 ***	-				
5. LE36	0.53 ***	0.37 ***	0.30 ***	0.28 ***	-			
6. LE48	0.36 ***	0.55 ***	0.39 ***	0.38 ***	0.60 ***	-		
7. LE60	0.31 ***	0.38 ***	0.51 ***	0.44 ***	0.50 ***	0.64 ***	-	
8. LE72	0.25 ***	0.32 ***	0.36 ***	0.55 ***	0.40 ***	0.56 ***	0.67 ***	-
*M*	17.13	19.03	20.45	21.67	30.05	32.98	34.38	34.96
*SD*	3.64	3.54	3.11	2.76	5.87	4.29	3.20	2.64
Min.	5	6	6	6	9	9	9	9
Max.	24	24	24	24	36	36	36	36

Note: EF = executive function; LE = language expression. Numbers indicate age in months. *** *p* < .001.

**Table 4:** Random intercept cross-lagged effects between EF and LE.

Category	Path	β	95% CI	*p*
Autoregressive	EF36 → EF48	0.25	[0.19, 0.32]	<.001
	EF48 → EF60	0.22	[0.15, 0.30]	<.001
	EF60 → EF72	0.09	[0.00, 0.18]	.042
	LE36 → LE48	0.45	[0.40, 0.50]	<.001
	LE48 → LE60	0.43	[0.37, 0.48]	<.001
	LE60 → LE72	0.42	[0.37, 0.47]	<.001
Concurrent	EF36 ↔ LE36	0.50	[0.46, 0.55]	<.001
	EF48 ↔ LE48	0.47	[0.42, 0.51]	<.001
	EF60 ↔ LE60	0.42	[0.36, 0.48]	<.001
	EF72 ↔ LE72	0.48	[0.41, 0.56]	<.001
Cross-lagged	EF36 → LE48	0.05	[−0.01, 0.10]	.095
	LE36 → EF48	0.16	[0.10, 0.22]	<.001
	EF48 → LE60	0.06	[0.00, 0.12]	.052
	LE48 → EF60	0.17	[0.10, 0.24]	<.001
	EF60 → LE72	0.00	[−0.08, 0.09]	.921
	LE60 → EF72	0.33	[0.25, 0.41]	<.001
Random intercept correlation	RI_EF ↔ RI_LE	0.55	[0.47, 0.63]	<.001

Note: EF = executive function; LE = language expression; RI_EF = random intercept of executive function; RI_LE = random intercept of language expression; β = STDYX standardized coefficient; CI = confidence interval. The 95% confidence intervals were obtained directly from the lower 2.5% and upper 2.5% values in the Mplus STDYX output.

## Data Availability

The data used in this study were obtained from the Kids in Taiwan: National Longitudinal Study of Child Development and Care (KIT), available through the Survey Research Data Archive, Academia Sinica. Restrictions apply to the availability of these data, which were used under license for the present study. Data are available from the Survey Research Data Archive upon application and approval.

## Abbreviations

The following abbreviations are used in this manuscript:

Abbreviation	Definition
CFA	Confirmatory factor analysis
EF	Executive function
FIML	Full information maximum likelihood
KIT	Kids in Taiwan: National Longitudinal Study of Child Development and Care
LE	language expression
RI-CLPM	Random intercept cross-lagged panel model

## Appendix A. Measurement Items

Executive Function Scale (6 items)

- The child can understand that his/her behavior may affect other people.
- When reminded, the child can slow down and do things better.
- The child can engage in cognitive or puzzle-based activities or games.
- When asked, the child can put toys or used objects back in their usual storage place.
- In public places, when asked to lower his/her voice, the child can immediately lower the volume and maintain it for at least several minutes.
- At home, when told “No,” the child can immediately stop running or jumping and maintain this for at least several minutes.

Language Expression Scale (9 items)

- The child can independently produce everyday descriptive words for people, events, or objects (e.g., “cold water,” “Auntie is pretty”).
- The child can respond to “What is this?” questions (e.g., when an adult asks “What is this thing?” the child answers “banana”; when asked “What place is this?” the child answers “school”).
- The child can respond to “Why?” questions (e.g., when an adult asks “Why aren’t you sleeping yet?” the child answers “I still want to play”).
- The child can produce complex sentences composed of two simple clauses (e.g., “The younger brother was naughty, so mother hit him”; “The injection hurt, but I did not cry”).
- The child can combine two simple sentences into one sentence using a connective (e.g., “because”) (e.g., “Because the younger brother was naughty, mother hit him”; “The injection hurt, but I did not cry”).
- The child can talk about people, events, or objects that are not currently present (e.g., if the teddy bear is not in the cradle, the child says, “The teddy bear is gone”).
- When talking with familiar people, the child can initiate a topic (e.g., when talking with parents or teachers).
- When the child cannot hear or understand something clearly, he/she can actively ask others to clarify (e.g., “What did you say?” “Can you say it again?”).
- The child can clearly explain the process of how to complete a task (e.g., explaining how to build a castle with blocks or explaining the rules of a game).
